# Evaluating the Global Plastic Waste Management System with Markov Chain Material Flow Analysis

**DOI:** 10.1021/acssuschemeng.2c04270

**Published:** 2023-01-30

**Authors:** Elijah Smith, Melissa M. Bilec, Vikas Khanna

**Affiliations:** † Department of Industrial Engineering, University of Pittsburgh, 3700 O’Hara Street, 1025 Benedum Hall, Pittsburgh, Pennsylvania15261, United States; ‡ Department of Civil and Environmental Engineering, University of Pittsburgh, 3700 O’Hara Street, 742 Benedum Hall, Pittsburgh, Pennsylvania15261, United States; § Department of Chemical and Petroleum Engineering, University of Pittsburgh, 3700 O’Hara Street, Pittsburgh, Pennsylvania15261, United States

**Keywords:** plastic waste, Markov chains, scenario modeling, recycling, waste trade

## Abstract

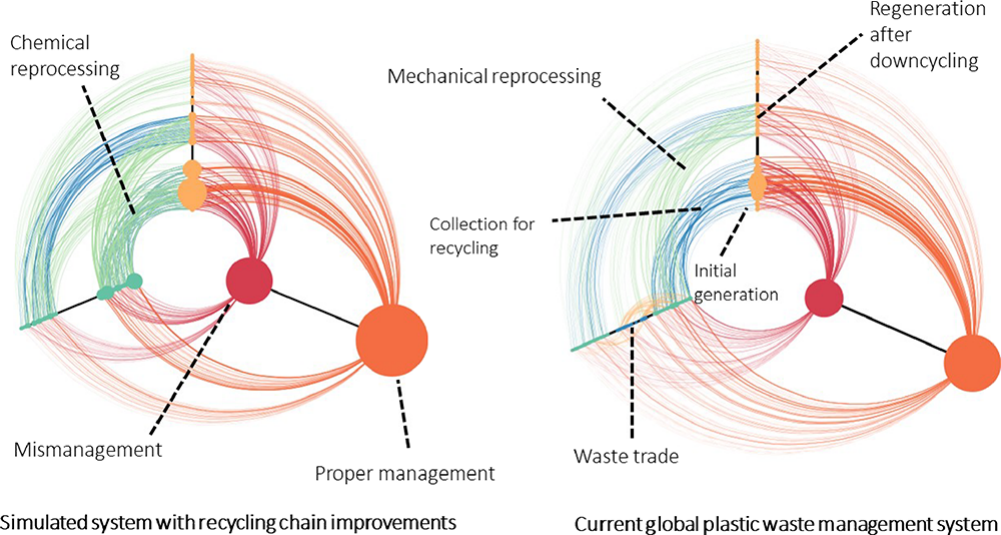

We present a global Markov chain-based material flow analysis of plastic waste of all types to estimate global virgin waste generation and waste mismanagement rates. We model nine alternative scenarios related to the elimination of plastic waste trade and improvements at various stages of the recycling chain, including “limitless” recycling promised by certain new chemical recycling technologies. We found that the elimination of trade increased global mismanagement when displaced waste was disposed but decreased mismanagement when it was instead recycled. Recycling scenarios showed little benefit for limitless recycling without prior increases in collection rates, which are currently the main constraint in the recycling chain. The most ambitious scenario only led to a 34% decrease in virgin waste generation. While significant, this implies that, given our current 40% mismanagement rate and 2050 forecasts of waste generation, landfilling and incineration capacity must increase 2.5-fold in addition to these extreme recycling targets to eliminate waste mismanagement. These results highlight the requirement for waste exporters to increase domestic recycling capacity as trade restrictions become tighter and express the urgent global need for alternative waste reduction interventions in addition to recycling.

## Abstract 2

We present a Markov chain model of global plastic waste management system and evaluate interventions to mitigate plastic pollution.

## Introduction

The world’s ever-increasing reliance on plastics has created an alarming environmental crisis, and its impact on human and ecological health is now too great to ignore. Five thousand eight hundred metric tons of primary plastic waste has been generated globally from 1950 through 2015, yet only 9% of that waste was recycled.^1^ Jambeck et al. estimated that, in 2010, 12.7 million metric tons of plastic waste entered the ocean, a number projected to increase by an order of magnitude by 2025 if no increases to plastic waste management capacity are made.^2^

Mismanaged plastic waste, defined by Jambeck et al. as waste that is littered or otherwise not formally managed, such as by disposal in dumps or open uncontrolled landfills,^2^ is of particular concern due to its high likelihood of entering the natural environment. More than 80% of marine plastic pollution enters through land-based sources, which highlights the detrimental impact of waste mismanagement. Mechanical degradation of plastics in the natural environment creates micro- and nanoplastics that are easily carried around the world via inland waterways, ocean currents, and wind.^3^ Marine species across the whole food web may consume microplastics, resulting in severe disruption to marine ecosystems.^4^ Many of these plastic particles are eventually ingested by humans, with a 2021 estimate finding that we likely consume between 0.1 and 5 g of plastic, which on the high end is equivalent to the weight of a credit card, each week.^5^ Although the full extent of the impacts of plastic waste on human health is poorly understood, recent research has demonstrated that plastic particles may act as pathogen vectors, and that plastic ingestion and inhalation pose a threat for particle and chemical toxicity induced by chemical additives, contaminants sorbed onto plastics, and the polymers themselves.^6^ Plastic waste mismanagement must therefore be mitigated to preserve ecological goods and services and human health.

The world’s collective plastic waste management infrastructure, which we denote the Global Plastic Waste Management System (GPWMS), is a complex networked system, consisting of many interconnected subsystems whose inputs and outputs simultaneously influence and are influenced by those of neighboring subsystems. The GPWMS can be described in multiple ways, but most broadly consists of three components. The first key component is virgin waste generation (VWG), which is where waste enters the system for the first time. Next is permanent disposal, which includes any type of waste management that renders the embedded polymers unrecoverable. This is the final fate of all plastics and can include landfilling, incineration (with or without energy recovery), littering, and open dumping. It includes both properly and improperly managed waste. Finally, there is everything else between the two, notably the full recycling chain and the waste trade network (PWTN), where the PWTN itself is just one subnetwork in the recycling chain. Additionally, recycling may be divided into waste collection, material recovery, and reprocessing, each of which taking many forms and utilizing a wide variety of technologies.

Plastic recycling is the focus of an immense body of scientific literature and is a large component of plastic pollution mitigation strategies across all levels of government. It undeniably will play a major role in the circular economy, yet many misconceptions remain, and its system level impact is still intensely debated. Only 9% of all plastic waste has been recycled, but no estimates exist on how much, if any at all, primary plastic production has been offset by recyclate.^1^ The uncertainty stems from the difficulty in estimating the displacement rate, which is the amount of VWG avoided due to the production of one unit of recyclate. Displacement is impacted by highly volatile economic factors that are difficult to measure and map to the consumer behaviors that determine displacement.^7^ It also cannot be measured directly due to its counterfactual nature, as it is an estimate of waste that was not produced, which is further explained by Zink et al.^7^ and Geyer et al.^1^

Additionally, the recycling chain is a complicated sequence of operations from waste collection to sorting and reprocessing, with each operation leaking some quantity of waste to permanent disposal. A variety of technologies are available to improve their efficiencies, but the potential at each step to reduce VWG globally has not been quantified. New chemical recycling technologies, some of which are purported to allow for infinite recycling with minimal degradation to recyclate quality, are nearly ready for mass implementation.^8,9^ Revealing the impact on VWG resulting from improved efficiencies for each component in the recycling chain, as well as their interaction effects, could identify the hotspots most in need of investment. At a higher level, an overall assessment of the capability of recycling to reduce waste under the most optimistic conditions may reveal its limitations and present a more accurate depiction of a mismanagement-free GPWMS.

Many smaller scale studies on recycling have been carried out to estimate process performance. Antonopoulos et al. estimated sorting and reprocessing efficiencies for several European recyclers and material recovery facilities (MRFs) using literature and primary sources.^10^ Jeswani et al. used the life cycle assessment framework to compare environmental impacts of chemical and mechanical recycling.^8^ Kuczenski and Geyer carried out a material flow analysis (MFA) of PET plastic from cradle-to-grave in the US, modeling the PET life cycle as a network of stocks and transformations.^11^ Importantly, they model recycling with flows from waste disposal back to production to allow for VWG displacement. They also consider trade, but only as a system input source and output sink for the US.

In recent years, the PWTN has fallen under the intense scrutiny of international policymakers, researchers, activists, and media alike. Many recyclers export a portion of their sorted plastic waste when they have insufficient capacity or demand for recyclate. Until 2018, China dominated the plastic waste import market, accounting for roughly half of all plastic waste imports by weight.^12^ However, after China implemented the National Sword Policy in 2018, their imports of postconsumer plastic waste fell to nearly zero.^13^ Key exporters in North America and Europe responded by shifting some of their waste exports elsewhere, with Southeast Asia taking in most of the displaced waste. Following their import surges, Malaysia, Vietnam, and Thailand responded with their own crackdowns on plastic waste imports.^14^

Brooks et al. confirmed that most plastic waste trade flows originate in high-income countries, where waste mismanagement is low, and end up in low or middle-income countries with less developed waste management infrastructure and comparatively lax environmental regulations.^12^ Consequently, the PWTN creates significant inequity, with some countries benefiting from it at the expense of others. It also has global implications for plastic pollution, and there is a need to understand the impacts of the waste trade on mismanagement to effectively curb plastic pollution.

While the need to understand the connection between plastic waste trade and mismanagement has been identified, no quantitative estimates exist that accurately determine the net impact of waste trade on plastic waste mismanagement. In fact, it would be impossible to assign just one value to it because there is no way of knowing how the evolution of the PWTN influenced the development of other waste management systems. If not traded, would the waste in the PWTN have been domestically recycled or landfilled? Did net importers increase recycling capacity purely to accept foreign waste, or did they decrease domestic recycling to accommodate foreign waste streams? How does the recyclate from imported waste impact domestic primary plastic generation? The present work does not aim to answer these questions, but the uncertainty in these areas highlights the need to study the PWTN within the broader context of the entire GPWMS.

The PWTN has been studied from a diverse set of perspectives. Pacini et al. analyzed the PWTN using a network model to characterize its general structure.^15^ Flow distributions were considered, and key participants were identified, but the results were not tied to any metrics of environmental impact. Most recent studies on the PWTN were motivated by China’s plastic waste import ban, which has left many researchers wondering about the fate of the displaced waste long-term, and how it will affect environmental health. Wen et al. used life cycle assessment to estimate the impact of the changes in trade flows using several environmental indicators.^16^ Liu et al. estimated net changes in greenhouse gasses resulting from China’s utilization of plastic waste imports before the ban.^17^ Brooks et al. used trade data to estimate that 111 million metric tons of plastic waste will be displaced by 2030 because of the import ban.^12^ Shi et al. used a network model of the PWTN and connected trade flow patterns to waste management policies in China.^18^

The import ban is clearly a well-studied topic, and a common thread between such works was the identification of huge amounts of waste with a highly uncertain future. Li et al. modeled the collapse of the PWTN as a cascading failure model due to the waste overloading experienced by several countries after the import ban.^17^ They found that the collapse of the network poses an ecological hazard, but scenario modeling showed that the risk was alleviated when countries prepared early by increasing their capacity. These analyses of the recent waste import ban give important insight into the dynamics of the system, but they do not estimate the impact on mismanagement of the PWTN as a whole, or rather, the potential impact of its complete elimination.

Here, we give the first estimates of the global impact of disruptions to the GPWMS involving the complete elimination of the PWTN. Our approach to elucidate this global impact of PWTN was to evaluate and compare the present GPWMS to four alternative scenarios (T.1–T.4, see details under Methods), representing the extreme ends of how the GPWMS could develop in the event of sudden elimination of plastic waste trade. We then estimated the net extra burden that trade places on each country’s management system to reveal the magnitude of the global inequity created by trade and used the comparative performance between the scenarios to identify key strategies that could help top plastic waste exporters prepare for trade bans. Additionally, we modeled five scenarios (R.1–R.5, see details under Methods) representing improved recycling systems to quantify how improved collection, sorting, and reprocessing efficiencies affect the GPWMS’s capacity to reduce permanent disposal rates, as well as their synergies. We considered scenarios with and without chemical recycling to estimate the potential benefit of theoretically “infinite” recycling. The recycling scenario results provide information needed to develop effective waste management strategies while also demonstrating the limitations of recycling as a standalone tool.

The goal of our present work is not to evaluate the absolute performance of the GPWMS but instead to examine the relative impact of eliminating plastic waste trade and implementation of recycling subsystems with respect to the baseline GPWMS. Furthermore, this work does not assess the potential impact of implementing any specific current or future recycling technologies (mechanical or chemical) for specific polymer types. Instead, the findings from this work should be viewed as capturing a broad range of possible relative outcomes that might result from plastic waste material reprocessing at a variety of sorting and reprocessing yields as well as recycling rates.

## Methods

### Markov Chain Model of the GPWMS

The core of analytical framework in the present work is the absorbing Markov chain. Absorbing Markov chains see frequent use in the study of stochastic processes and in recent years have been used in the field of industrial ecology for MFA.^19^ Prior works that use Markov chain material flow analysis (MCMFA) focused primarily on metals, such as aluminum,^20^ nickel,^19^ copper,^21^ and steel,^22^ which are particularly suited to this type of model due to their near infinite recyclability. MCMFA has seen use for other materials too, such as e-waste in Indonesia^23^ and clothing.^24^ These models all consider life cycle stages before waste generation, focusing on the use phase, whereas we only consider the life cycle stages from post first use to final absorption into the natural environment. For a theoretical overview of MCMFA, its underlying assumptions, and how it differs from common stock-and-flow models, see Eckelman et al.’s MCMFA of nickel.^19^ The Assumptions section in Methods further discusses several key distinctions between this work and other MCMFA models, particularly with how time was handled.

To model the GPWMS, we represented the state of some arbitrary quantity of plastic waste as an absorbing discrete-time Markov chain, where the state space captures its country-level geographic location and life cycle stage from initial waste generation through ultimate disposal into the natural environment. We did not define the duration of a time-step because we were not interested in time-based performance metrics and assumed transition probabilities to be static, which is further explained in the Assumptions section. The transition probabilities therefore represented the probability of a waste transition from state *i* to state *j* once it transitions.

For ease of reference, we partitioned the state space into the following subsets:

G: Generation of waste. These are origin nodes, i.e., the entry points of plastic waste in the model.
R: Plastic in recovery systems (MRFs, chemical and mechanical recycling).

These were systems who output recyclate usable for the production of new plastic products. We combined material recovery and both chemical and mechanical recycling systems into one state to reduce computation times, instead modeling their individual yield as a multiplier for transitions out of this state. That is, transitions out of R depended on the MRF yield as well as chemical and mechanical recycling proportions and their respective yields. The difference in how chemical and mechanical recycling were modeled is described below in Methods.

I: Imported waste. Globally, over half of waste collected for recycling was exported in 2017, so plastic waste trade must be modeled. Imported waste was assumed to be sorted by the exporter, which necessitated higher import recycling rates than domestic recycling rates. Because of this, we created states for imported waste, rather than allowing traded waste to flow directly between R states.

Mismanaged (MM): Plastic that was improperly managed. This is a dummy node used to represent the absorption point of mismanaged waste in the system.

Managed properly (MP): Plastic that was properly managed. This is a dummy node used to represent the absorption point of properly managed waste in the system.

The states G, R, and I are sets of states whose elements represent a point in the plastic waste life cycle for a specific country. So, G_*i*_ refers to waste generation in country *i*, *i* = 1,2, ..., *n* where *n* is the number of countries. Flows from G to one of the sinks (MM or MP) represent permanent disposal of waste without any attempt at recovery. Transitions from G to R represent plastic collected for recycling, and a transition from R back to G means that the waste was successfully recovered and converted to usable recyclate. We left out the use and reuse life cycle stages and instead rerouted waste directly back to generation, to the same effect, as we assumed all plastic goods used in country *i* must have transitioned to G_*i*_ before moving to any other state. Flows from R to I represent waste exports.

#### Modeling Mechanical vs Chemical Recycling

**Figure 1:**
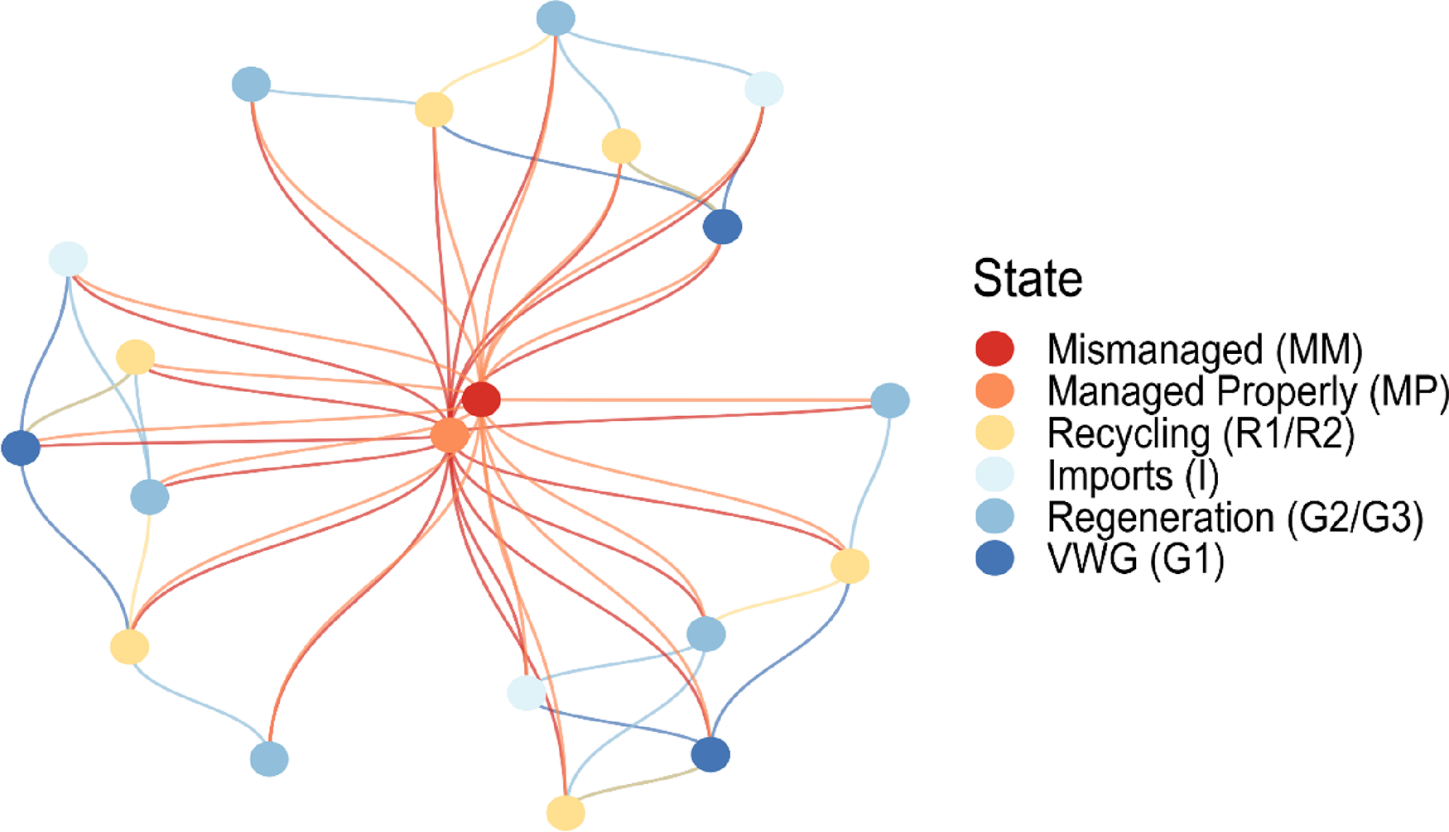
Example schematic of Markov chain structure for three countries. Trade flows are not shown. Edges are colored by the destination state. The complete model structure has 141 countries.

The above model would incorrectly assume infinite recyclability of plastics because waste can transition from G to R and back to G infinitely many times. In reality, mechanical recycling typically degrades the quality of plastic waste that limits the number of times plastic may be recycled. The severity of this degradation is dependent on a number of complicated factors such as polymer type, additives, and the sorting and reprocessing technology.^25^ To account for this degradation, we assumed that waste may be mechanically recycled no more than twice as low overall recycling rates make it exceedingly unlikely that waste ever returns to a recycling facility for the third time. To encode this limit in the model while maintaining the above structure, the transition probabilities from R to G would be dependent on the number of times that the waste has already visited R, which would violate the Markov property. We circumvented this issue by expanding the state space such that states G and R become G1, G2, G3, R1, and R2. Waste thus enters the model at G1 and will go to R1 if recycled. It can then transition back to G1 if chemically recycled, G2 or G3 if mechanically recycled for the first or second time, or one of the sinks (MM or MP) if not successfully recovered. With this adjustment, plastic waste can only be mechanically recycled two times successively before it must be disposed of permanently. Figure 1 below shows a three-country component of the Markov chain model. The full model is an assembly of such subnetworks for all countries (141 in total), with trade flows connecting them at R and I.

### Transition Probabilities

The transition probabilities in this work are computed from empirical data where possible but often must be randomly sampled.

From G1, transition probabilities to R1 are determined by the recycling rate. Transitions to the absorbing states are calculated as the product of “1–recycling rate” and management/mismanagement rates. G2 and G3 transition probabilities are all computed similarly, with the one change that mechanical recycling rates at G3 are zero.

From R1, waste can transition to either G1, G2, I (in every other country), or the sinks. Transitions back to G1 occur with probability equal to the product of “1–trade fraction,” sorting efficiency, chemical recycling fraction, and chemical reprocessing efficiency. Transitions from R1 to G2 are computed similarly, substituting the latter two values with their mechanical recycling counterparts. Transitions from R_*i*_ to I_*j*_ occur with probability equal to the probability of waste being exported from I_*i*_ to R_*j*_ given that it is exported. All remaining waste transitions to the sinks according to their mismanagement rates.

From I, waste can transition to G1 with a probability that is the product of import recycling rate, chemical recycling fraction, and chemical reprocessing efficiency, and transitions to G2 are the same as the former but with the mechanical recycling counterparts. The remaining portion goes to the sinks, allocated according to the import mismanagement rates.

The final states are the sinks, MM and MP. Because they are absorbing states, they are defined to transition back to themselves with a probability of 1, and with a probability of 0 to all other states.

### Data

Data used in this study was from a variety of sources. Where data was absent, we used values across time periods and also applied the results of studies on European and American waste management practices to other countries, using relatively wide intervals to sample unknown values for Monte Carlo simulation. Total waste generation (TWG), mismanagement, import recycling, and import mismanagement rates were extracted from the work of Law et al., who estimated them using the 2016 World Bank report on country-level municipal solid waste practices and composition studies of waste outputs of European material recovery facilities.^26^ The same World Bank report provides MSW recycling rates, which we found to have no relationship with plastic recycling. We found plastic recycling rates for 20 countries from multiple other sources and recycling rates for the remaining countries were sampled from a uniform distribution. Sorting and reprocessing efficiencies were sampled from probability distributions as well as based on studies of European facilities.^10^ Plastic waste trade data was obtained from the CEPII-BACI database,^27^ which uses UN Comtrade data^28^ and attempts to reconcile the differences in trade flows as reported by importers and exporters based on their reporting reliability. Section S1 in the Supporting Information provides all the data sources used and discusses our methodology for translating the results of studies into parameter values for use in our present study.

In total, five unknown parameters are used to compute transition probabilities, which were sampled from underlying probability distributions in Table 1. For a single iteration, we sampled one global value for each parameter to be used for every country. If separate samples were used for each country, it would be expected that around half of the countries would have values toward the lower end of the distribution and half toward the higher end, so their variances would, to some degree, “cancel” each other out every iteration, and the total variance in our calculations of the global performance of the system (described in Evaluation of the GPWMS subsection below) between iterations would be underestimated.

**Table 1:** Transition Probability Distributions for Monte Carlo Simulation and Their Respective Data Sources

parameter	distribution	source
recycling rate^a^	uniform (0.05, 0.15)	multiple, see Table S8 in the Supporting Information
sorting efficiency	uniform (0.59, 0.83)	(10)
reprocessing efficiency	uniform (0.70, 0.85)	(10)
import recycling rate^b^	uniform (0.75, 0.85)	(26)
import mismanagement rate^c^	uniform (0.25, 0.75)	(26)

a Only for countries with unknown recycling rates.

b For all but 11 countries, who required an additional country-specific upper limit due to low or negative VWG due to recycling offset. The cap was such that imported waste recyclate may not offset more than 10% of any country’s VWG. See the Supporting Information for methodological details.

c For countries with greater than 20% mismanagement. Import mismanagement was zero for other countries.

### Evaluation of the GPWMS

We calculated the global performance of the baseline and each alternative by the total mismanaged waste (TMW) and virgin waste content (VWG/TWG) that they resulted in. We started by calculating VWG, which is the rate that waste enters the system through each G1_*i*_. This must also be the rate at which waste leaves the system in steady state; if this was not the case, the total quantity of waste in the system would either tend toward zero or infinity as time passed. Waste was considered to have left the system the instant it reaches one of the sinks.

The transition matrix of the Markov chain model allowed us to relate the throughput of every state to the input rates at G1, which can be used in conjunction with TWG data to construct a system of linear equations that compute VWG rates. We first found the throughput at each element of G1, G2, and G3 for one unit of input at each element of G1. By rearranging the transition matrix *P* such that transient states (all states excluding MM and MP) occurred first, we partition *P* as:
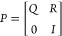
1*Q* contains the transition probabilities between transient states, *R* contains the transition probabilities from each transient to each absorbing state, 0 is a matrix of zeroes indicating that no transitions occur from absorbing to transient states, and *I* is an identity matrix indicating that absorbing states always transition back to themselves. We may now define the matrix *N* such that:
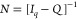
2*I_q_* is an identity matrix of the same dimension as *Q*, and *N* is called the fundamental matrix. Each element (*n_ij_*) of *N* can be interpreted as the expected throughput at state *j* when one unit of material is supplied to state *i*. We can now write the equation to calculate TWG for one country *i* as:
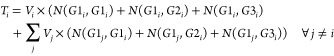
3where *T_i_* and *V_i_* are the TWG and VWG for country *i*. Matrix *N* can be partitioned as follows:
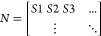
4where *S*1, *S*2, and *S*3 are all *n* × *n* matrices giving the throughputs at G1, G2, and G3 for each country *i* = 1, 2, ..., n. Eq 3 can be rewritten in the matrix form as:
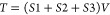
5and
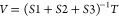
6

Absorption probabilities then be calculated. The absorption probabilities between the states in G1 and MM indicate the expected proportion of waste that will be mismanaged given its country of origin. If we define the vector *R*′ to be the column of *R* corresponding to MM, then the desired absorption probabilities *A*′ can be calculated as
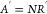
7

We truncate *A*′ to only include absorption probabilities for states in G1 to obtain the vector *A*″. Finally, we calculate *M_T_*, the total quantity of mismanaged waste created by the system, as:
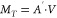
8

The elements of *M_T_* do not represent the total waste mismanaged within each country’s borders, but rather the total mismanaged waste that each country is responsible for. As an example, if country *i* produces a unit of virgin waste, exports it to *j*, and country *j* then mismanages it, we say that country *i* is responsible for that mismanaged waste as opposed to country *j*.

The above metrics are useful for judging global performance but do not reveal the disparities between countries, which is critical for the evaluation of the PWTN. As a measure of the net burden that the PWTN places on each country, we calculated the percent change in total waste disposed (TDW) within each country’s borders between the baseline GPWMS and several no-trade alternatives. The TDW for country *i* is the quantity of waste that saw final permanent disposal within country *i*’s borders. To calculate it, we first computed the total waste disposed from each state *S_k_* as:
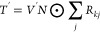
9where *V*′ is a row vector containing the input rates for each transient state (i.e., 0 for all states not in G1 and *V_i_* for each country *i* in G1), ∑*_j_R_kj_* is a column vector where each element *k* gives the probability that waste at state *S_k_* transitions to one of the sink states in its immediate next step, and *v* ⊙ *w* denotes pairwise vector multiplication of *v* and *w*. *T*′ is therefore the rate at which waste flows from each state to the sinks. TDW is then calculated by grouping the elements of *T*′ by country and taking their sum. Note that TDW is linear with respect to TMW, so an *x*% reduction in TDW results in an *x*% reduction in TMW.

#### Assumptions

##### Aggregation of Waste Types and Recycling Technologies

We treated all waste types and recycling technologies as equivalent in all respects. As a result, this work may not be interpreted as an assessment of any current recycling technologies but instead quantifies the impact of material reprocessing in general. The distinction between chemical and mechanical recycling only shows the theoretical benefit of the additional reprocessing cycles after the first two permitted by mechanical recycling. We made these estimates at a variety of sorting and reprocessing yields as well as recycling rates so as to reflect a variety of possible implementations of chemical depolymerization rather than focusing on specific types. A polymer type also impacts recycling yields, but we ignore the distinction between them for the same reason. Additionally, many types of chemical recycling produce fuels rather than new plastic products. For the purposes of this study, “recycling” only refers to processes that create polymer recyclate that displace virgin plastic products, and any other waste reprocessing falls under permanent disposal.

##### Displacement of Virgin Waste

We assumed 100% displacement between recyclate and virgin plastic, and that displacement occurred instantaneously. Life cycle assessment (LCA) studies typically make the same assumption, but it rarely holds true. The displacement rate is decided by market forces, making it notoriously difficult to estimate.^7^ In the absence of data to estimate the displacement rate, we assumed it equal to one and made a subtle change in the interpretation of the performance metrics. We considered the displacement rate to lie outside the scope of the GPWMS, because even though it does impact waste flows, it is highly volatile and of a completely different nature than the other parts of our model, which reflect material services and industrial processes instead of an abstract market force. Instead of representing actual performance, the metrics in our work reflected the best performance that the GPWMS could produce given its infrastructure, ignoring the short-term effect on displacement of consumer preferences between primary and secondary plastics.

Our assumption that mechanical recycling happens no more than twice is invalid for many polymers (notably PET) and recycling processes. However, this had a negligible impact on the results as the benefit of recycling decreases geometrically with respect to the number of times waste has been recycled. To illustrate this, consider a simple example of one country with no trade and only chemical recycling. Waste enters at the state G and then can either transition to the recycling state R or a sink state S. From R, waste can either return to G if recovered, or transition to S otherwise. For simplicity, let *r* denote the probability of transitioning from G to R and back to G, making *r* the joint probability of collection for recycling, successful sorting, and successful reprocessing.

For one unit of input to G, VWG displaced by the first recycling cycle is equal to *r*. This waste ends up back at G, where the proportion *r* of that waste will also be recycled and return to G. So with two recycling cycles, the displacement equals *r* + *r*^2^. With infinite recycling, this translated to the following series.
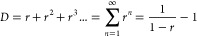
10where *D* is the amount of recyclate produced for one unit of input. This geometric series reflects infinite recycling, while mechanical recycling with a limit of two cycles truncates the series after the *r*^2^ term. Note that for the baseline scenarios, all values of *r* were extremely low. For example, the US was assumed to have an average sorting efficiency of 71%, reprocessing efficiency of 77.5%, and recycling rate of 9%, resulting in *r* = 0.05. The geometric series therefore converges rapidly, with the first two terms *r* + *r*^2^ = 0.0525, and the full series *D* = 0.0526. Thus, the difference in displacement between chemical and mechanical recycling is *D* – (*r* + *r*^2^), or 0.0001 for the US. This suggests that the theoretical benefit of infinite recycling is very low with current recycling rates, and that our decision to model mechanical recycling as a maximum of two cycles had little impact, as the *r*^2^ term and everything after it were both very small. However, the geometric series converges at a slower rate as *r* grows, so chemical recycling has a much larger displacement with higher recycling collection rates. With a 50% recycling rate, the infinite series equals 0.68, while the first two terms sum to 0.57. This simple model ignores trade, which would be quite complicated to take into consideration to estimate values for *r_i_* for countries *i* = 1, 2, ..., N. However, if *r_i_* is displacement for country *i* assuming no trade, and *q_i_* is displacement permitting exports, then *r_i_* ≥ *q_i_* if countries have similar *r_i_* values due to imports incurring an additional loss in recovered material from the import recycle rate. Therefore, our simple no-trade model places an upper bound on true displacement, making the above analysis more robust in the presence of trade.

##### Markov Chain Model Assumptions

Our model does not define the size of a time step, instead assuming that waste flows continuously through the system at a steady rate. The transition probability *P_ij_* gives the proportion of waste at state *i* that transitions to state *j* once it does transition. We therefore have no self-loops, i.e., transitions from *i* to *j* where *i* = *j*, and we assume that waste flows continuously through the system in steady state. Considering time as in dynamic MFA or several other MCMFA models is only beneficial where time-based performance metrics and the historical behavior of the system are of interest, or where transition probabilities themselves are dynamic. For example, Eckelman et al. used MCMFA to estimate the technological lifetime of nickel with a cradle-to-grave model.^19^ Like ours, their model did not define a universal duration to a time step, so they first estimated the average number of visits to each state and the average time spent at each, which were used to estimate the time spent between ore extraction and permanent disposal. Approaches that explicitly model stocks, such as Kuczenski and Geyer’s MFA of PET plastic, continuously track material over some duration and are useful to illustrate the historical evolution of a system.^11^

We ignored time between transitions and treated transition probabilities as static, which may pose an issue for two main reasons. First, it may take a long time for waste to move from G1 to G3, over which time transition probabilities may change. This could have been modeled by using different probabilities for transitions from G1 to R1 and G2 to R2, but in absence of accurate country-level forecasts for recycling rates and estimates of how long it takes to move from, say, G1 to G2, we assumed recycling rates to be constant. The other issue relates to time of displacement and time of permanent disposal. Waste produced in 1 year might be recycled later, ultimately displacing virgin waste the next year. Similarly, waste may enter the system as VWG in 1 year but not see permanent disposal until much later. So, VWG was calculated using TWG for just 1 year but displacement for the full waste life cycle. Because of these assumptions, our VWG estimates may not reflect the true waste inputs for any given year, but instead give the input VWG rate required to sustain a particular TWG rate in the long run.

### Scenario Modeling

Due to the large uncertainty for most transition probabilities, we used Monte Carlo simulation with 10,000 iterations for scenario modeling. Unknown parameters were sampled pairwise for all scenarios and then used in conjunction with point estimates of the remaining parameters to create transition matrices. That is, for a given iteration, one value was sampled for each parameter and applied to all countries for all scenarios, allowing pairwise comparisons between scenario performance.

From the transition matrices, we calculated VWG rates and absorption probabilities for each country, which were used to calculate TMW produced by the global system. We then found the difference in VWG and TMW between the baseline and each alternative scenario to map changes in subsystem parameters to changes in global performance. We would like to emphasize that, unlike most MFAs, we were not interested in the absolute performance for alternatives or baselines. The absolute results considered flows for the full GPWMS, including impacts from both the modified flows and the rest of the GPWMS. Instead, our objective was to evaluate the impact of the trade and recycling subsystems within the context of the full GPWMS, which was achieved by considering the difference between each scenario and the baselines.

Subtracting out the baselines from the alternatives and using virgin content rather than VWG also served to mitigate uncertainty in TWG values, which we treated as point estimates. TWG was constant across all scenarios, and because of its linear relationship with VWG, virgin content did not depend on TWG when recycling parameters were equal for each country. This assumption was only minimally violated in the baselines, trade scenarios, and R.1 through R.4, and not at all for R.5. Virgin content therefore has very little dependence on TWG and is highly robust to data uncertainty. We modeled the baseline GPWMS along with nine alternative scenarios and an additional baseline with slightly different assumptions, described in table 2. The first four alternatives reflect the elimination of waste trade (T.1–T.4), and the last five (R.1–R.5) all relate to improved recycling systems.

**Table 2:** Description of Baseline and Alternative Scenario Models that Were Assessed via Monte Carlo Simulation^a^

scenario	description
(B.1) baseline, accepted import recycling rates	our model of the current system with most likely values for import recycling rates, which are the minimum of the sampled value given in Table 1 and the country-specific maximum required to avoid extremely low or negative VWG values
(B.2) alternative baseline, 20% import recycling rate maximum	alternative baseline model. Pessimistically assumed that importers recycle no more than 20% of their imports. It was not intended to be a reasonable representation of the current system, but comparing it with the no trade scenarios revealed reasonable worst case scenario impacts of the PWTN
(T.1) elimination of PWTN, displaced waste landfilled	trade fractions were set to zero, recycling (collection) rates reduced such that all waste that would have been traded was landfilled before reaching R
(T.2) elimination of PWTN, displaced waste recycled	trade fractions were set to zero, and recycling rates were kept the same so that all waste that would have been traded was domestically recycled
(T.3) elimination of PWTN, displaced waste is landfilled, excess recycling capacity is used	trade fractions are set to zero, recycling rates for exporters were reduced such that all waste that would have been traded is landfilled before reaching R. Recycling rates were then increased for importers such that recycling capacity that would have processed foreign waste was instead used to process domestic waste. Net exporters were left with reduced recycling collection rates, while net importers saw increased recycling collection
(T.4) elimination of PWTN, displaced waste is recycled, excess recycling capacity is used	trade fractions were set to zero, recycling rates for importers were increased such that recycling capacity that would have processed foreign waste was instead used to process domestic waste
(R.1) increase sorting efficiency	sorting efficiency was set to 90%
(R.2) increase sorting and reprocessing efficiency	sorting and reprocessing efficiency were both set to 90%
(R.3) 50% recycling rate, all mechanical	all countries achieved 50% recycling rates, all new recycling capacity was mechanical, trade fractions were set to zero
(R.4) 50% recycling rate, some chemical	all countries achieved 50% recycling rates, 75% of new recycling capacity was chemical, trade fractions were set to zero
(R.5) 50% recycling, some chemical, increase sorting and reprocessing	this was the most optimistic recycling scenario, combining (R.2) and (R.4). Achieving this by 2050 is unlikely, but including this overly ambitious scenario served to set proper expectations regarding the effectiveness of recycling

a Scenarios B.1 and B.2 represent baselines, with B.2 reflecting pessimistic assumptions regarding recycling of imported waste. Trade scenarios are compared to both baselines, while recycling scenarios only consider B.1. Scenarios T.*X* reflect zero trade scenarios, and scenarios R.*X* all reflect improvements along the recycling chain. Plastic waste trade network = PWTN.

## Results and Discussion

### Waste Trade Scenarios

**Figure 2:**
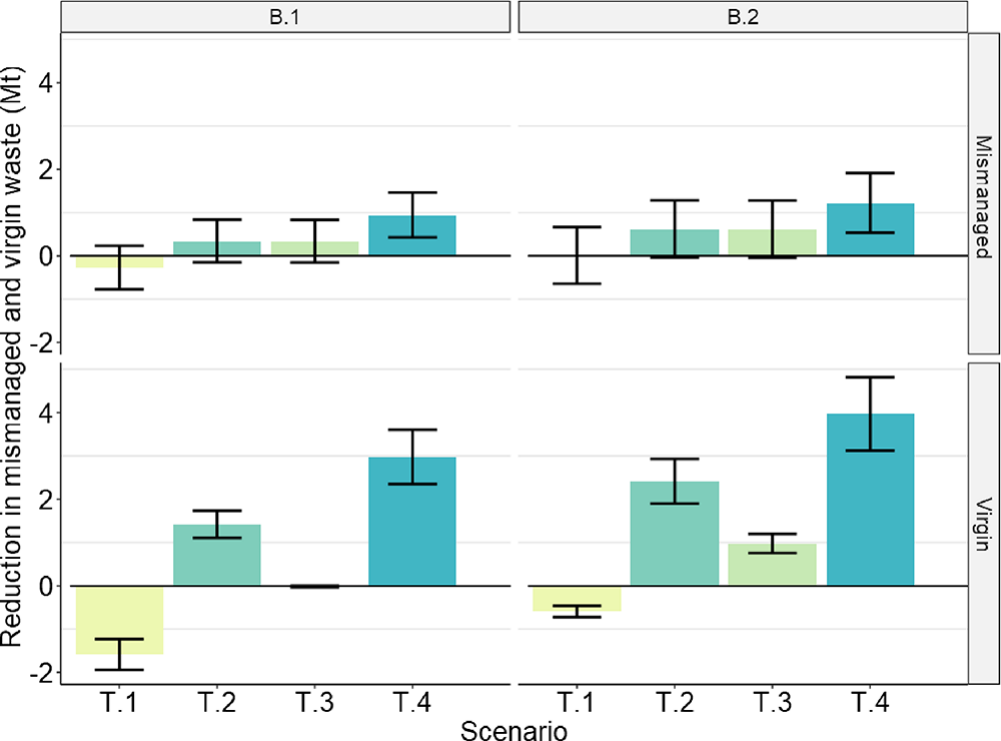
Global impact of trade elimination scenarios (T.l) displaced waste landfilled, (T.2) displaced waste recycled, (T.3) displaced waste landfilled, excess capacity used, (T.4) displaced waste recycled, excess capacity used against the baselines B.1 and B.2 on total mismanaged and virgin waste under both baselines. See Table 2 for scenario descriptions. Positive values indicate that eliminating trade reduced TMW and/or VWG under that scenario, while negative values indicate that eliminating trade increased global TMW and/or VWG. Error bars show two standard deviations of distance from the mean.

Figure 2 shows the results for plastic waste mismanagement and virgin waste generation under various trade scenarios for the GPWMS. The results in Figure 2 are plotted relative to the two baseline scenarios (B.1. and B.2). In the best case, eliminating trade reduced mismanagement by 1.2 Mt and VWG by 4.0 Mt (scenario T.4 under baseline B.2). In the worst case (scenario T.1 under baseline B.1), eliminating trade increased mismanagement and VWG by 0.3 and 1.6 Mt, respectively.

It may be surprising that trade could have a positive impact considering that importers tend to have higher mismanagement rates than exporters, but this occasionally occurred when eliminating trade decreased overall global recycling rates, particularly in high mismanagement countries. For example, with scenarios T.1 and T.3, net exporters choose to permanently dispose the extra waste that they may no longer trade, although in the baseline, we assumed that some of that waste was recycled. This shifted waste from recycling facilities typically in high mismanagement importing countries to landfills and incinerators in low mismanagement countries, leading to increased VWG for the importers. Scenarios T.2 and T.4 instead shift the displaced waste to recycling facilities in the exporting countries, which prevents the drop in overall recycling but still leads to increased VWG among importers with a decrease for exporters.

Both T.1 and T.2 imply that net importers lack the collection and transportation infrastructure to supply the recycling facilities currently managing imported waste, which we assume leads to their closure after losing imported waste streams. Scenarios T.3 and T.4 imply that importers can supply them to keep recycling rates up, allowing them to utilize the excess recycling capacity for their domestic waste. Taken together, these scenarios demonstrate that trade restrictions in the future could have negative consequences if exporters increase permanent disposal and importers do not make use of the excess recycling capacity. Efforts to reduce waste trade should therefore go beyond simply stopping trade flows by also pushing for increased domestic recycling.

The implication that stopping waste trade may lead to overall increased mismanagement is highly relevant considering China’s waste import ban. The policy created a global shock that led to a rapid restructuring of the PWTN and the broader GPWMS, therefore providing a real example for how the system responds to sudden shocks. From this, we can better understand which of the trade scenarios are more likely. So far, most have found that the import ban increased domestic waste management, but not recycling. Thus, exporters for the most part have chosen to either increase permanent disposal or find new trade partners, rather than increase recycling. However, such studies have also mentioned that an increase in permanent disposal was expected in the short term, with recycling increases only coming later if at all. Our results demonstrate that increasing domestic recycling is critical for certain top exporters.

**Figure 3:**
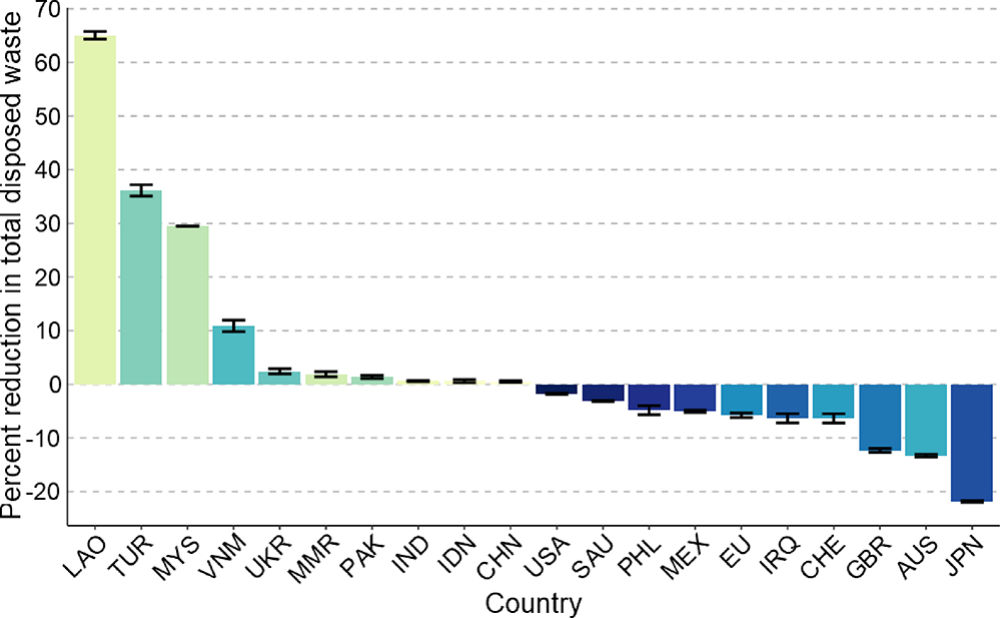
Percent reduction in total waste disposed within each country’s border resulting from eliminating trade according to scenario T.1, relative to baseline B.1. See Table 2 for scenario description. Positive values indicate that eliminating trade reduced the quantity of waste disposed within the country’s borders, and thus reflect a positive incentive to halt trade. The first 10 countries are the top net importers with >20% mismanagement (accounting for 49% of imports), and the last 10 are the top net exporters (accounting for 77% of exports). Error bars show a coverage of two standard deviations.

**Figure 4:**
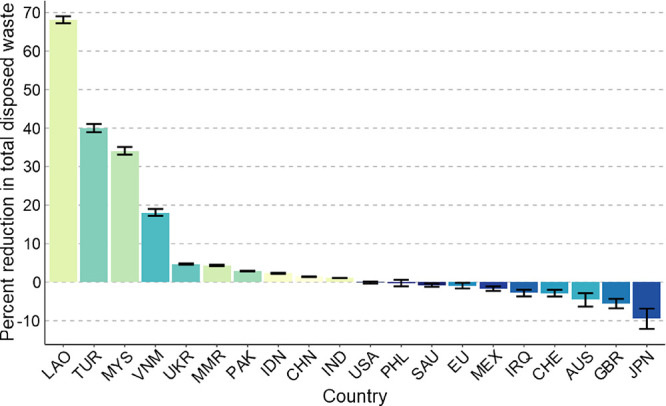
Percent reduction in total waste disposed within each country’s border resulting from eliminating trade according to scenario T.4, relative to baseline B.1. See Table 2 for scenario description. Positive values indicate that eliminating trade reduced the quantity of waste disposed within the country’s borders, and thus reflect a positive incentive to halt trade. The first 10 countries are the top net importers with >20% mismanagement (accounting for 49% of imports), and the last 10 are the top net exporters (accounting for 77% of exports). Error bars show a coverage of two standard deviations.

Figure 3 shows the percentage reduction in total disposed waste that trade elimination has on select countries in the GPWMS relative to the baseline scenario under scenario T.1, where net exporters bear the largest negative impact due to displaced trade flows. At the country level, we observed tremendous disparities in the net burden trade places on each country’s waste management system across all scenarios. The results in Figure 3 are shown for the top 10 importers with greater than 20% mismanagement rates, and the top 10exporters. Figure 4 shows the same quantities for scenario T.4 instead, where net exporters recycle displaced waste and net importers use the excess capacity. These two scenarios lie at extreme ends and show the range of country-level impacts that could result from waste trade stoppages, which highlights the significance of the concomitant waste management policy changes resulting from trade stoppages.

**Figure 5:**
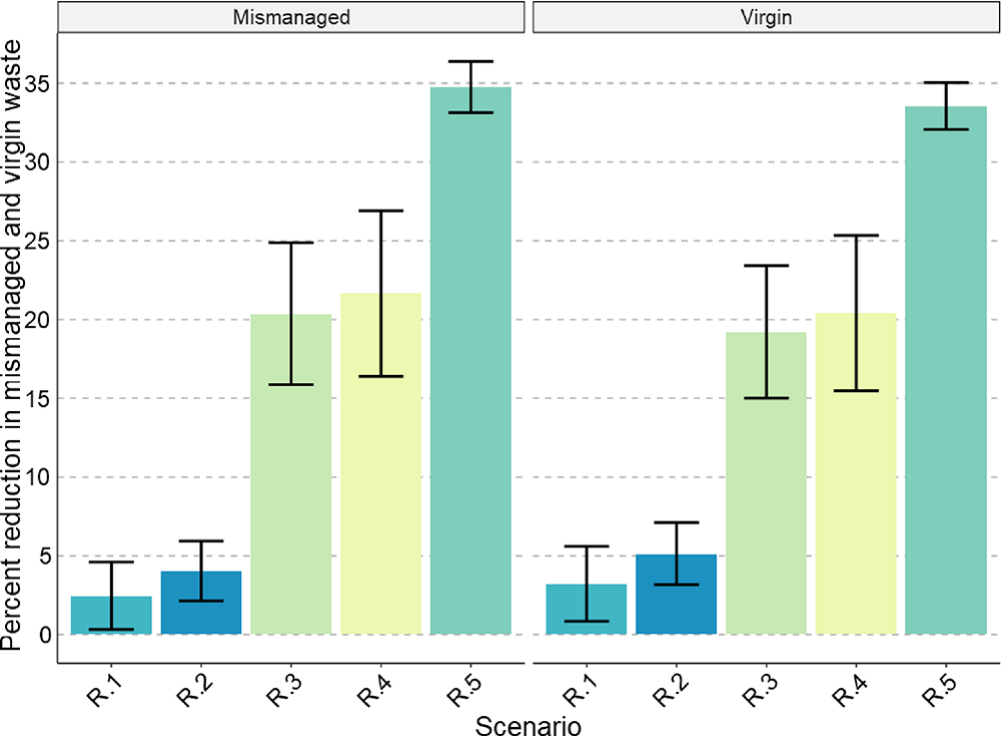
Global percent reductions in TMW and VWG for the improved recycling scenarios (R.1) increase sorting efficiency, (R.2) increase sorting and reprocessing efficiency, (R.3) 50% recycling rate, all mechanical, (R.4) 50% recycling rate, some chemical, and (R.5) 50% recycling, some chemical, increase sorting and reprocessing efficiency. See Table 2 for scenario description. Bar height gives the percent reduction in waste (mismanaged or virgin) for the scenarios relative to baseline (B.1). The reductions are calculated by subtracting the scenario outcome from the baseline, dividing by the baseline outcome, and multiplying by 100. Error bars show 2 standard deviations of distance from the mean.

Laos, for example, saw an average 65% reduction in the amount of waste disposed within their borders by eliminating trade, whereas Japan saw a 22% increase. A few of the top importers see larger reductions in waste disposed when using excess recycling capacity shown in Figure 5 for scenario T.4, particularly Malaysia and Vietnam, but for many, it makes little difference if they use the excess capacity or not. This is because many of the top importers are assumed to be disposing (or perhaps re-exporting) most of the imported waste, leaving them with little excess capacity spent on imported waste. We may be overestimating import disposal rates for those countries, but others who do recycle much of their imported waste, for example Vietnam, Pakistan, and Malaysia, demonstrate that stopping waste imports to increase recycling of domestic waste may significantly reduce a country’s waste management burden. As such, we can generally conclude that top importers have a strong incentive to halt imports and benefit from doing so regardless of if they use the excess capacity, and that those who do recycle much of their imports see an even greater benefit from reducing or eliminating trade when they are able to use the excess recycling capacity. This can only be accomplished if they have the collection, transportation, and sorting infrastructure required to supply these facilities with steady waste streams. If they do not at the time of restricting imports, the recycling facilities might shut down, leading to increased VWG. Importers should therefore evaluate their waste management systems and identify actions necessary to supply these facilities with sorted waste prior to stopping imports.

Several of the top exporters saw a significant extra burden placed on their waste management system after eliminating trade. The top five most impacted countries, and their worst-case increase in TDW, were Japan (22%), Australia (13%), The UK (12%), Switzerland (10%), and the Philippines (8%). These countries find themselves in a precarious position; by relieving significant pressure on their management systems through trade, they could see a damaging shock to their systems if they suddenly lost the ability to export. Other top exporters (notably the US, Saudi Arabia, and the EU) hardly registered a change when they stopped trading. This occurred because top exporters generally produced huge quantities of waste that overshadowed their exports.

When the top 10 exporters recycled the displaced waste instead, the extra burden was reduced by a factor of 2–3 relative to T.1 with landfilling of displaced waste. Recycling the displaced exports therefore can greatly reduce the potential shock to their systems, making exporters more robust to sudden changes in waste trade policy. Many other countries not mentioned here are greatly impacted by trade as well. The waste trade is dominated by a handful of key players, but the smaller players typically produce much less waste and therefore may see large relative impacts. Tables S6 and S7 in the Supporting Information show how each country was impacted by the loss of trade in each scenario relative to both baselines B.1 and B.2, respectively.

Because the burden that trade places on top importers exceeds the relief on the management systems of exporters, there subjectively appears to be more pressure against trade than in favor of it, at the national policy level at least. This is consistent with the recent developments in the PWTN, where importers have been implementing strict import regulations and exporters have been slowing their export.^13^ Economic incentives may drive exporters to seek out new trade partners after Southeast Asia stops accepting waste as was observed after China’s import ban,^13^ but so far, most countries have been quick to intervene in response to import spikes, forcing exporters to domestically manage some of the displaced exports. This, along with growing international collaboration to mitigate the harms of waste trade and stronger government interventions in waste management in the developing world, may suggest that plastic waste trade may continue this downward trend.

### Improved Recycling Scenarios

Figure 5 presents the results for the global percent reductions in TMW and VWG resulting from improvements along the recycling chain. These scenarios demonstrated that substantial reductions in VWG are possible with recycling infrastructure improvements. In the most optimistic scenario (scenario R.5), VWG equals 60.9% ± 0.1% of TWG, compared to 91.3% ± 0.2% for the baseline B.1. With today’s consumption patterns, such VWG values correspond to a minimum global permanent disposal capacity of 199.0 ± 4.5 Mt for the baseline, and 132.7 ± 0.27 for the most optimistic recycling scenario.

The recycling scenarios also showed that collection rates have a large impact on VWG and should be prioritized over technical improvements to the effectiveness of sorting and reprocessing operations (although the two are not mutually exclusive of course). Improved collection rates yielded larger improvements because they are much farther from their theoretical maxima than sorting and reprocessing efficiencies. The end-of-life recycling rate controls VWG as a proportion of TWG and is the product of recycling rate, sorting efficiency, and reprocessing efficiency. Because of this relationship, changes in any of those factors result in the same impact on the end-of-life recycling rate when that change is expressed multiplicatively. Thus, if a country has a 10% recycling rate and an 80% sorting efficiency, increasing their recycling rate to 11% would yield the same reduction in VWG as increasing their sorting efficiency to 88%. This also explains why scenario R.5 has a greater impact than the sum of R.4 and R.2. Increasing sorting and reprocessing efficiencies has a much larger impact when collection rates are higher, as increased collection allows more waste to take advantage of the greater efficiencies. These synergistic effects should be taken advantage of in the design of recycling policies. For example, collection schemes that better separate waste early in the recycling chain will make both sorting and reprocessing more efficient due to the higher quality inputs, therefore displacing more VWG than an equivalent increase in single-stream waste collection.

These scenarios revealed that, while recycling has significant potential to reduce VWG, it can in no way solve the plastic problem alone. In the most optimistic scenario with global recycling rates of 50%, efficiency of 81%, and widespread adoption of chemical recycling technology, virgin content was only reduced by around 30%. This corresponds to a virgin content of 60% if displacement between recyclate and primary plastic is one-to-one. Even if these targets are achieved by 2050, which would be an astounding accomplishment requiring strong government initiatives, immense investment, and international cooperation across the design, manufacture, consumption, and waste management sectors, permanent disposal rates will remain unchanged if waste generation continues to grow as projected. Considering that roughly 40% of waste ends up mismanaged today, stopping all mismanagement by 2050 would require that permanent disposal capacity sees a 60% increase in addition to those ambitious recycling targets.

It would be difficult to overstate the severity of this issue. A global recycling rate of 50% is projected for 2050,^1^ but this makes much less of a difference unless sorting and reprocessing efficiencies increase dramatically as well. Technological improvements can help accomplish this, but the quality of input waste streams contributes heavily to recycling efficiency.^10^ An 81% recycling efficiency would therefore require better collection systems, product designs compatible with material recovery, and reduced use of composite materials and additives like dyes and binders, as well as ubiquitous implementation of state-of-the-art technology. The low and lower-middle income countries who make up most of the projected growth in waste generation only collect 40 and 50% of the waste that they produce respectively, meaning that they must largely build these advanced recycling systems from the ground up.^29^

On top of that, chemical recycling is still in its infancy and likely will not see the mass adoption by 2050 assumed by the most optimistic scenario, and recyclate does not offset primary plastic one-to-one. Low displacement rates would erase most of the benefits of recycling, so building new recycling infrastructure must occur in parallel with incentivizing the consumption of secondary plastics. Certain chemical recycling technologies tackle both issues at once by producing high-quality recyclate with properties that are theoretically identical to those of primary plastic, whereas mechanical recycling degrades the material and outputs a product that is typically of inferior quality or suitable only for lower value applications. In addition to matching the quality of primary plastics, recyclate must be cheaper than raw materials. Large-scale investment in the recycling infrastructure therefore may have little impact unless it occurs simultaneous with policies like recycling subsidies,^30^ virgin plastic taxes,^31^ or other EPR policies^32^ that drive up the cost of primary plastics.

## Conclusions

We present a country-level Markov chain material flow model of the global plastic waste management system across the full waste life cycle, from virgin generation to final disposal. At the global level, two baselines and nine alternative scenarios were modeled, reflecting changes to the PWTN and recycling systems. The GPWMS was then evaluated for each scenario in terms of VWG and TMW. Finally, we compared the performance of alternative and baseline scenarios to estimate the impacts of changes to the PWTN and recycling subsystems. Additionally, we performed an additional country-level analysis to estimate the net burden that waste trade places on each country. Trade scenario results showed that eliminating waste trade can have either a positive or negative impact, depending on how the rest of the GPWMS adapts. Country-level results indicated that waste trade places high pressure on several net importers, who may soon stop imports. Exporters may see significant shocks to their system if they lose trade partners, which can be reduced by a factor of 2 to 3 through increased domestic recycling. Recycling scenarios revealed that collection rates are the most important, as improvements in efficiency or the use of infinite chemical recycling technologies make a very little difference at current recycling levels. However, the benefits were shown to grow with better collection rates. The most optimistic recycling scenario only resulted in a 34% virgin content, so landfilling and incineration capacity must increase by a factor of 2.5 in addition to the most optimistic recycling improvement scenarios to eliminate plastic waste mismanagement by 2050, if waste generation continues to grow as projected.

## Supporting Information Available

Data sources and methods and definitions and tables about reduction in waste (Mt) and (%) no trade scenarios, reduction in waste (Mt) and (%) improved recycling scenarios, percent reduction in total disposed waste for baseline B.1 and B.2, expected virgin content (VWG/TWG) improved recycling scenarios, and plastic recycling rate data sources (PDF)

## Abbreviations

GPWMS: Global Plastic Waste Management System
MCMFA: Markov chain material flow analysis
MFA: material flow analysis
MM: mismanaged
MP: managed properly
MRF: material recovery facility
PWTN: plastic waste trade network
TDW: total waste disposed
TMW: total mismanaged waste
TWG: total waste generation
VWG: virgin waste generation
