# Supplementary material for: Evaluating the Global Plastic Waste Management System with Markov Chain Material Flow Analysis
Source: ACS Sustain Chem Eng. 2023 Jan 30;11(6):2055–65. doi: 10.1021/acssuschemeng.2c04270 (PMC10034734; doi:10.1021/acssuschemeng.2c04270)
Supplement: Supplementary file 1 — sc2c04270_si_001.pdf [file sc2c04270_si_001.pdf]

“Supporting Information” for:

Evaluating the global plastic waste management system with Markov chain material flow analysis

Elijah Smith[1], Melissa M. Bilec[2], and Vikas Khanna[2,3]*

[1] Department of Industrial Engineering, University of Pittsburgh, 3700 O’Hara Street, 1025 Benedum Hall, Pittsburgh, Pennsylvania 15261, United States

[2] Department of Civil and Environmental Engineering, University of Pittsburgh, 3700 O’Hara Street, 742 Benedum Hall, Pittsburgh, Pennsylvania 15261, United States

[3] Department of Chemical and Petroleum Engineering, University of Pittsburgh, 3700 O’Hara Street, Pittsburgh, Pennsylvania 15261, United States

*Address correspondence to khannav@pitt.edu; telephone: +1-412-624-9604

Number of pages: 16
Number of figures: None
Number of tables: 9

S1 – Data sources and methods

We use waste mismanagement rates come from Law et. al., who estimated them using the 2016 World Bank report on country-level municipal solid waste practices and composition studies of waste outputs of European material recovery facilities.[1] These mismanagement rates assume that recycling is a final disposal option (i.e., a mutually exclusive alternative to mismanagement), but our model treats recycling as an intermediary stage. We therefore must adjust the mismanagement rates to instead represent the proportion of linearly disposed waste that is mismanaged, which is accomplished by dividing the mismanagement rates by the term (1 – recycling rate).

Total waste generation rates come from the same World Bank report [2], but we use Law's refined estimate for the US's plastic waste generation. We use point estimates for these values, but the World Bank emphasizes the unreliability of these estimates. Rather than subjectively assigning probability distributions, we report performance metrics as a proportion of TWG where appropriate.

Recycling rate data was available for 20 countries from a variety of data sets shown in table S10, (with the EU member states aggregated into one country for all analyses), so recycling rates must be randomly generated for most countries. Many countries report recycling rates for specific types of waste such as PET bottles or packaging, but this waste is inherently easier to recycle and more often collected than other common types of plastic waste. We therefore do not use these values to estimate general plastic recycling. The World Bank estimates municipal solid waste recycling rates for most countries, but we found no relationship between these and reported plastic recycling rates. We therefore assume that all other recycling rates fall between 5% and 15% based on UN Center for Regional Development publications that subjectively claim that lower income countries (who make up the overwhelming majority of the countries with unknown recycling rates) tend to have recycling rates comparable to the US's 9% as well as the other published rates that suggest that this is a reasonable range.

Sorting and reprocessing efficiency rates come from Antonopoulos et al., who use primary and literature data from European material recovery and recycling facilities to estimate these values.[3] Their analyses showed high variance in these values between facilities, so we sample these values from a probability distribution rather than using point estimates. We use a uniform distribution with bounds equal to the mean plus/minus the 95% confidence bounds.

Trade data comes from the CEPII-BACI database. BACI data is originally sourced from the UN Comtrade database, which provides plastic waste trade flows reported by both the exporter and importer. These values should be identical, but often are not. CEPII reconciles these differences by taking the average of the importer and exporter reports weighted by each country's reporting reliability. See the CEPII-BACI working paper for the detailed explanation of how reliability is calculated.[4] We use 2019 trade data for commodities with HS92 codes beginning with 3915, which is all types of plastic waste. To calculate the trade fraction, we divide each country's total exports by their total waste collected for recycling. For 16 countries, exports exceed total waste collected for recycling, with some even having exports that exceed the sum of TWG and imports. To reconcile this mass imbalance, we set a maximum trade fraction of 0.9, which led to an 8.7% reduction in trade network throughput.

Import recycling and mismanagement rates for most countries were determined in the same way as Law et. al. Their analysis of composition studies of plastic waste bales leaving material recovery facilities in Europe revealed that they contain approximately 75-85% material valuable to recyclers. They also assumed that 25-75% of disposed imported waste was mismanaged in countries with greater than 20% mismanagement rates, and 0% in the remaining countries.[1]

With this assumption, we found that several countries had extremely low and sometimes negative VWG rates. This is clearly impossible but occurred in the model because VWG was estimated via a set of unbound linear equations. These abnormalities only affected top importers who were recycling quantities of imported waste approaching or exceeding their TWG. These unrealistic VWG values provide strong evidence that key importers recycle a much smaller proportion of their imported waste than the 75-85% that we originally assumed. We applied a correction to import recycling rates such that imports can offset no more than 10% of VWG, which is generous but reasonable relative to other countries in the baseline model (who typically have a virgin content upwards of 90%). The rest of the waste is assumed to be disposed. The max import recycling rates were determined as so:

For each country, let I be the quantity of imports, T their TWG, d the VWG offset by imports as a proportion of T, r_I the import recycling rate, and r_e the end-of-life recycling rate excluding exports. The value of d may be expressed as

$$d = \frac{I(r_I + r_I r)}{T} = \frac{I r_I (1+r)}{T} \leq 0.1 \quad (1)$$

which gives

$$r_I = min\left\{r_S, \frac{0.1T}{I(1+r)}\right\} \quad (2)$$

where r_s is the import recycling rate randomly sampled according to the original assumptions. The cap affected eleven countries who were collectively responsible for 2% of TWG and 52% of imports and led to a global 65% reduction in import recycling rates.

See table S9 for a tabulated display of the sources, along with a detailed list of recycling rate sources in table S8.

Table S1 – Reduction in waste (Mt), no trade scenarios. This data corresponds to figure 2 in the article.

Baseline	Waste type	Scenario	Scenario code	Mean	Standard deviation	Lower coverage bound	Upper coverage bound
B.1	Mismanaged	no trade, landfill	T.1	-0.267	0.251	-0.769	0.235
B.1	Mismanaged	no trade, recycle	T.2	0.343	0.247	-0.151	0.836
B.1	Mismanaged	no trade, landfill, use capacity	T.3	0.339	0.247	-0.155	0.832
B.1	Mismanaged	no trade, recycle, use capacity	T.4	0.945	0.259	0.427	1.464

B.1	Virgin	no trade, landfill	T.1	-1.587	0.177	-1.942	-1.232
B.1	Virgin	no trade, recycle	T.2	1.420	0.156	1.108	1.732
B.1	Virgin	no trade, landfill, use capacity	T.3	-0.015	0.008	-0.031	0.001
B.1	Virgin	no trade, recycle, use capacity	T.4	2.978	0.313	2.352	3.604
B.2	Mismanaged	no trade, landfill	T.1	0.013	0.327	-0.642	0.667
B.2	Mismanaged	no trade, recycle	T.2	0.622	0.330	-0.038	1.283
B.2	Mismanaged	no trade, landfill, use capacity	T.3	0.618	0.331	-0.043	1.279
B.2	Mismanaged	no trade, recycle, use capacity	T.4	1.225	0.346	0.533	1.916
B.2	Virgin	no trade, landfill	T.1	-0.593	0.065	-0.723	-0.464
B.2	Virgin	no trade, recycle	T.2	2.414	0.258	1.899	2.929
B.2	Virgin	no trade, landfill, use capacity	T.3	0.978	0.111	0.757	1.200
B.2	Virgin	no trade, recycle, use capacity	T.4	3.971	0.422	3.127	4.816

Table S2 – Reduction in waste (%), no trade scenarios.

Baseline	Waste type	Scenario	Scenario code	Mean	Standard deviation	Lower coverage bound	Upper coverage bound
B.1	Mismanaged	no trade, landfill	T.1	-0.318	0.299	-0.917	0.281
B.1	Mismanaged	no trade, recycle	T.2	0.406	0.292	-0.178	0.991
B.1	Mismanaged	no trade, landfill, use capacity	T.3	0.402	0.292	-0.182	0.985
B.1	Mismanaged	no trade, recycle, use capacity	T.4	1.123	0.308	0.507	1.739
B.1	Virgin	no trade, landfill	T.1	-0.795	0.097	-0.989	-0.602
B.1	Virgin	no trade, recycle	T.2	0.712	0.085	0.541	0.882
B.1	Virgin	no trade, landfill, use capacity	T.3	-0.008	0.004	-0.016	0.000
B.1	Virgin	no trade, recycle, use capacity	T.4	1.492	0.172	1.148	1.836
B.2	Mismanaged	no trade, landfill	T.1	0.013	0.387	-0.762	0.788
B.2	Mismanaged	no trade, recycle	T.2	0.736	0.389	-0.042	1.514
B.2	Mismanaged	no trade, landfill, use capacity	T.3	0.731	0.389	-0.048	1.510

B.2	Mismanaged	no trade, recycle, use capacity	T.4	1.450	0.408	0.633	2.266
B.2	Virgin	no trade, landfill	T.1	-0.296	0.035	-0.366	-0.225
B.2	Virgin	no trade, recycle	T.2	1.204	0.140	0.924	1.484
B.2	Virgin	no trade, landfill, use capacity	T.3	0.488	0.059	0.369	0.607
B.2	Virgin	no trade, recycle, use capacity	T.4	1.980	0.229	1.521	2.439

Table S3 – Reduction in waste (Mt), improved recycling scenarios.

Scenario	Scenario code	Waste type	Mean	Lower coverage bound	Upper coverage bound
increase sorting	R.1	Mismanaged	2.08E+06	9.14E+05	2.55E+05
increase sorting & yield	R.2	Mismanaged	3.40E+06	8.27E+05	1.75E+06
50%, mechanical	R.3	Mismanaged	1.71E+07	1.77E+06	1.36E+07
50%, chemical	R.4	Mismanaged	1.82E+07	2.07E+06	1.41E+07
increase mech, chem, yield	R.5	Mismanaged	2.93E+07	9.19E+05	2.74E+07
increase sorting	R.1	Virgin	6.47E+06	2.43E+06	1.62E+06
increase sorting & yield	R.2	Virgin	1.03E+07	2.06E+06	6.16E+06
50%, mechanical	R.3	Virgin	3.83E+07	3.85E+06	3.07E+07
50%, chemical	R.4	Virgin	4.07E+07	4.54E+06	3.17E+07
increase mech, chem, yield	R.5	Virgin	6.71E+07	2.22E+06	6.26E+07

Table S4 – Reduction in waste (%), improved recycling scenarios. This data corresponds to figure 5 in the article

Scenario	Scenario code	Waste type	Mean
increase sorting	R.1	Mismanaged	2.47
increase sorting & yield	R.2	Mismanaged	4.04
50%, mechanical	R.3	Mismanaged	20.37
50%, chemical	R.4	Mismanaged	21.65

increase mech, chem, yield	R.5	Mismanaged	34.75
increase sorting	R.1	Virgin	3.23
increase sorting & yield	R.2	Virgin	5.14
50%, mechanical	R.3	Virgin	19.21
50%, chemical	R.4	Virgin	20.42
increase mech, chem, yield	R.5	Virgin	33.56

Table S5 – Percent reduction in total disposed waste for baseline B.1. Positive values indicate a reduction in TDW when trade is eliminated. Uncertainty is expressed as two standard deviations of distance from the mean.

	**Mean**				**Uncertainty**			
Country	T.1	T.2	T.3	T.4	T.1	T.2	T.3	T.4
LAO	64.2	66.1	66.4	68.2	0.9	0.3	0.4	1.5
TUR	36.4	36.8	40.3	40.7	0.6	0.5	2.3	2.3
VGB	27.6	27.6	32	32	0.9	0.9	1.9	1.9
MYS	25	26	35.6	36.5	1.5	1.2	1.4	1.7
UZB	16.5	16.5	21.6	21.7	1.4	1.4	1.9	1.9
HKG	16.3	22.4	28.4	33.8	2.5	0.7	1	2.3
TWN	12.9	14.9	18.2	20.2	1.7	1.4	1.8	2
VNM	11.1	13.5	16.5	18.9	2	1.8	1.5	1.7
CAN	9.3	13.5	13.9	18	1.1	0	0.2	1.3
SRB	4.1	4.6	7.9	8.4	1	0.8	0.4	0.4
NOR	3.2	8.4	9	14.1	8.3	4.9	4.6	2.1
BIH	2.7	5.8	8	11	2.4	2	0.4	0.9
UKR	2.4	2.6	4.6	4.8	0.6	0.5	0.2	0.2
KOR	1.9	2.4	4.2	4.6	0.5	0.4	0.2	0.3
MMR	1.8	2.3	4	4.4	0.5	0.4	0.1	0.2
AZE	1.7	1.8	3.2	3.2	0.4	0.4	0.1	0.1
HND	1.7	2.8	4.9	6	1	0.8	0.7	0.8
PAK	1.4	1.5	2.7	2.9	0.3	0.3	0.1	0.1
SEN	1.3	2.5	4.2	5.3	0.7	0.4	0.1	0.4
GIN	0.8	0.8	1.4	1.4	0.2	0.2	0	0
BGD	0.6	0.8	1.5	1.6	0.2	0.2	0.1	0.1
CHN	0.6	0.8	1.3	1.6	0.2	0.1	0.1	0.1
IDN	0.6	1.1	2.1	2.6	0.3	0.2	0.1	0.2
IND	0.6	0.6	1.1	1.1	0.1	0.1	0	0
MAR	0.5	1	1.7	2.2	0.3	0.1	0.1	0.2
BTN	0.4	0.5	0.9	0.9	0.1	0.1	0	0
LKA	0.4	0.5	0.9	1	0.1	0.1	0	0

OMN	0.4	1.1	1.8	2.5	0.4	0.2	0.1	0.2
PER	0.4	0.5	0.9	1	0.1	0.1	0.1	0.1
PRY	0.4	0.7	1.1	1.4	0.2	0.1	0	0.1
ZAF	0.3	0.4	0.8	0.9	0.1	0.1	0	0.1
NAM	0.2	0.2	0.4	0.4	0	0	0	0
VUT	0.2	0.2	0.3	0.3	0	0	0	0
ECU	0.1	0.6	1	1.5	0.2	0.1	0.1	0.2
EGY	0.1	0.1	0.2	0.2	0	0	0	0
NGA	0.1	0.3	0.5	0.8	0.1	0.1	0	0.1
AND	0	0	0	0	0	0	0	0
ARG	0	0	0	0	0	0	0	0
BEN	0	0	0.1	0.1	0	0	0	0
BMU	0	0	0.1	0.1	0	0	0	0
CIV	0	0.1	0.1	0.2	0	0	0	0
COD	0	0	0	0	0	0	0	0
COG	0	0	0	0	0	0	0	0
CUB	0	0	0	0	0	0	0	0
DMA	0	0	0	0	0	0	0	0
FRO	0	0	0	0	0	0	0	0
GRD	0	0	0	0	0	0	0	0
GUM	0	0	0	0	0	0	0	0
LCA	0	0	0.1	0.1	0	0	0	0
MDA	0	0	0	0	0	0	0	0
MDG	0	0	0	0	0	0	0	0
MNP	0	0	0	0	0	0	0	0
NER	0	0	0	0	0	0	0	0
NPL	0	0	0.1	0.1	0	0	0	0
PNG	0	0	0	0	0	0	0	0
PSE	0	0	0	0	0	0	0	0
PYF	0	0	0	0	0	0	0	0
RUS	0	0.2	0.3	0.5	0.1	0	0	0.1
SLB	0	0	0	0	0	0	0	0
SMR	0	0	0	0	0	0	0	0
SYR	0	0	0	0	0	0	0	0
TON	0	0	0	0	0	0	0	0
TUV	0	0	0	0	0	0	0	0
UGA	0	0	0	0	0	0	0	0
WSM	0	0	0	0	0	0	0	0
BRA	-0.1	0	-0.1	0	0	0	0	0
DZA	-0.1	-0.1	-0.1	0	0	0	0	0
MAC	-0.1	0.4	0.5	1	0.1	0	0	0.1
COL	-0.2	-0.1	-0.2	0	0	0	0	0.1
HTI	-0.3	-0.1	-0.3	-0.1	0	0	0	0
JOR	-0.3	-0.1	-0.3	-0.1	0	0	0	0
MNG	-0.3	-0.1	-0.3	-0.1	0	0	0	0

ZWE	-0.3	0	-0.1	0.2	0	0	0	0.1
BOL	-0.4	-0.1	-0.4	-0.1	0	0.1	0	0.1
CYM	-0.4	0	-0.1	0.3	0.1	0	0	0.1
KEN	-0.4	-0.1	-0.3	0	0	0.1	0	0.1
MNE	-0.4	0	-0.1	0.4	0.1	0	0	0.1
TGO	-0.4	-0.1	-0.3	-0.1	0	0	0	0.1
TTO	-0.4	-0.1	-0.3	-0.1	0	0	0	0.1
TZA	-0.4	-0.1	-0.2	0.1	0	0	0	0.1
ARM	-0.5	-0.1	-0.4	0	0	0.1	0	0.1
BFA	-0.5	-0.2	-0.5	-0.2	0	0.1	0	0.1
KNA	-0.6	-0.3	-0.6	-0.3	0	0.1	0	0.1
SUR	-0.6	-0.3	-0.6	-0.2	0	0.1	0	0.1
URY	-0.6	-0.1	-0.3	0.1	0.1	0.1	0	0.1
YEM	-0.6	0.8	0.9	2.3	0.4	0.1	0	0.4
GUY	-0.7	-0.3	-0.7	-0.3	0	0.1	0	0.1
KAZ	-0.7	-0.3	-0.6	-0.2	0	0.1	0	0.1
BWA	-0.8	-0.2	-0.5	0.1	0.1	0.1	0	0.2
CMR	-0.8	-0.3	-0.7	-0.2	0	0.1	0	0.1
MRT	-0.8	-0.3	-0.7	-0.2	0	0.1	0	0.1
IRN	-1	-0.4	-1	-0.4	0	0.1	0	0.1
QAT	-1	-0.3	-0.8	-0.1	0.1	0.1	0	0.2
BRN	-1.1	-0.3	-0.8	-0.1	0.1	0.1	0	0.2
SDN	-1.1	-0.5	-1.1	-0.5	0	0.2	0	0.2
ABW	-1.2	-0.5	-1.2	-0.5	0	0.2	0	0.2
ARE	-1.2	0.1	-0.3	1	0.3	0.1	0	0.3
ETH	-1.3	-0.5	-1.2	-0.5	0.1	0.2	0	0.2
MOZ	-1.3	-0.2	-0.7	0.4	0.2	0.1	0	0.3
CHL	-1.4	-0.5	-1.3	-0.4	0.1	0.2	0	0.2
KGZ	-1.4	-0.5	-1.3	-0.4	0.1	0.2	0	0.2
TJK	-1.4	-0.4	-1.1	-0.1	0.1	0.2	0	0.2
BHR	-1.5	-0.4	-1.2	-0.1	0.1	0.2	0	0.3
GTM	-1.6	-0.5	-1.2	-0.1	0.1	0.2	0	0.3
PAN	-1.7	-0.7	-1.6	-0.6	0.1	0.2	0.1	0.3
USA	-1.8	-0.5	-1.2	0.1	0.1	0.2	0	0.4
DOM	-2.2	-0.9	-2.1	-0.7	0.1	0.3	0.1	0.4
LBN	-2.2	-0.9	-2	-0.7	0.1	0.3	0.1	0.3
ISR	-2.6	-0.9	-2.2	-0.5	0.1	0.3	0.1	0.4
KWT	-2.6	-1.1	-2.5	-1.1	0.1	0.4	0.1	0.4
CUW	-3	-1.3	-3	-1.3	0.1	0.4	0.1	0.4
SAU	-3.1	-1.1	-2.7	-0.7	0.1	0.4	0	0.5
BLR	-3.2	-0.5	-1.9	0.7	0.4	0.4	0.1	0.7
THA	-3.3	0	-1	2.3	0.7	0.3	0	0.9
MUS	-3.6	-1.3	-3.2	-0.9	0.2	0.5	0.1	0.6
MDV	-3.7	-1.3	-3.2	-0.8	0.2	0.5	0.1	0.6
SLV	-3.7	0.1	-1.1	2.6	1.1	0.6	1	1.1

BRB	-4	-1.8	-4	-1.8	0.1	0.6	0.1	0.6
BLZ	-4.1	-1.8	-4.1	-1.8	0.1	0.6	0.1	0.6
SGP	-4.2	-1.2	-3.2	-0.3	0.3	0.5	0.1	0.7
GRL	-4.7	-2.1	-4.7	-2.1	0.2	0.7	0.2	0.7
MEX	-5	-2	-4.6	-1.6	0.2	0.6	0.1	0.7
EU	-5.7	-1.8	-4.3	-0.4	0.6	0.6	0.2	0.9
NIC	-6.3	-2.5	-5.9	-2.1	1	1	1	1.1
MKD	-6.6	-2.1	-5.4	-0.9	2.4	1.5	2.4	1.7
VCT	-6.6	-2.7	-6.4	-2.5	1.3	1.2	1.3	1.2
IRQ	-7.4	-3.2	-7.4	-3.2	1.9	1.5	1.9	1.5
PHL	-7.8	-2.6	-6.5	-1.3	4.2	2.3	4.2	2.4
TUN	-8.1	-3.4	-7.9	-3.2	2.9	1.9	2.9	1.9
FJI	-8.2	-3.3	-7.7	-2.8	3.1	1.8	3.1	1.9
KHM	-8.8	-3.6	-8.4	-3.3	4.1	2.4	4.1	2.4
NZL	-8.9	-3.2	-7.7	-2.1	5.9	3	5.9	3.1
JAM	-9	-3.9	-8.9	-3.9	3.8	2.3	3.8	2.3
CRI	-9.3	-3.4	-8.3	-2.4	6.7	3.4	6.6	3.5
CHE	-9.9	-4.4	-9.9	-4.3	5.3	3	5.3	3
ISL	-10.7	-4.7	-10.7	-4.7	6.9	3.6	6.9	3.6
MHL	-10.7	-4.7	-10.7	-4.7	6.9	3.6	6.9	3.6
TKM	-10.7	-4.7	-10.7	-4.7	6.9	3.6	6.9	3.6
GBR	-12.3	-5.6	-12.2	-5.4	0.5	1.4	0.4	1.5
AUS	-13.2	-5.2	-12.3	-4.2	0.3	1.8	0.1	2
JPN	-21.8	-9.5	-21.5	-9.2	0.2	3	0.1	3.1

Table S6 – Percent reduction in total disposed waste for baseline B.2. Positive values indicate a reduction in TDW when trade is eliminated. Uncertainty is expressed as two standard deviations of distance from the mean.

	**Mean**				**Uncertainty**			
Country	T.1	T.2	T.3	T.4	T.1	T.2	T.3	T.4
LAO	64.2	66.1	66.4	68.2	0.9	0.3	0.4	1.5
TUR	36.4	36.8	40.3	40.7	0.6	0.5	2.3	2.3
VGB	27.6	27.6	32	32	0.9	0.9	1.9	1.9
MYS	25	26	35.6	36.5	1.5	1.2	1.4	1.7
UZB	16.5	16.5	21.6	21.7	1.4	1.4	1.9	1.9
HKG	16.3	22.4	28.4	33.8	2.5	0.7	1	2.3
TWN	12.9	14.9	18.2	20.2	1.7	1.4	1.8	2
VNM	11.1	13.5	16.5	18.9	2	1.8	1.5	1.7
CAN	9.3	13.5	13.9	18	1.1	0	0.2	1.3
SRB	4.1	4.6	7.9	8.4	1	0.8	0.4	0.4
NOR	3.2	8.4	9	14.1	8.3	4.9	4.6	2.1
BIH	2.7	5.8	8	11	2.4	2	0.4	0.9
UKR	2.4	2.6	4.6	4.8	0.6	0.5	0.2	0.2
KOR	1.9	2.4	4.2	4.6	0.5	0.4	0.2	0.3

MMR	1.8	2.3	4	4.4	0.5	0.4	0.1	0.2
AZE	1.7	1.8	3.2	3.2	0.4	0.4	0.1	0.1
HND	1.7	2.8	4.9	6	1	0.8	0.7	0.8
PAK	1.4	1.5	2.7	2.9	0.3	0.3	0.1	0.1
SEN	1.3	2.5	4.2	5.3	0.7	0.4	0.1	0.4
GIN	0.8	0.8	1.4	1.4	0.2	0.2	0	0
BGD	0.6	0.8	1.5	1.6	0.2	0.2	0.1	0.1
CHN	0.6	0.8	1.3	1.6	0.2	0.1	0.1	0.1
IDN	0.6	1.1	2.1	2.6	0.3	0.2	0.1	0.2
IND	0.6	0.6	1.1	1.1	0.1	0.1	0	0
MAR	0.5	1	1.7	2.2	0.3	0.1	0.1	0.2
BTN	0.4	0.5	0.9	0.9	0.1	0.1	0	0
LKA	0.4	0.5	0.9	1	0.1	0.1	0	0
OMN	0.4	1.1	1.8	2.5	0.4	0.2	0.1	0.2
PER	0.4	0.5	0.9	1	0.1	0.1	0.1	0.1
PRY	0.4	0.7	1.1	1.4	0.2	0.1	0	0.1
ZAF	0.3	0.4	0.8	0.9	0.1	0.1	0	0.1
NAM	0.2	0.2	0.4	0.4	0	0	0	0
VUT	0.2	0.2	0.3	0.3	0	0	0	0
ECU	0.1	0.6	1	1.5	0.2	0.1	0.1	0.2
EGY	0.1	0.1	0.2	0.2	0	0	0	0
NGA	0.1	0.3	0.5	0.8	0.1	0.1	0	0.1
AND	0	0	0	0	0	0	0	0
ARG	0	0	0	0	0	0	0	0
BEN	0	0	0.1	0.1	0	0	0	0
BMU	0	0	0.1	0.1	0	0	0	0
CIV	0	0.1	0.1	0.2	0	0	0	0
COD	0	0	0	0	0	0	0	0
COG	0	0	0	0	0	0	0	0
CUB	0	0	0	0	0	0	0	0
DMA	0	0	0	0	0	0	0	0
FRO	0	0	0	0	0	0	0	0
GRD	0	0	0	0	0	0	0	0
GUM	0	0	0	0	0	0	0	0
LCA	0	0	0.1	0.1	0	0	0	0
MDA	0	0	0	0	0	0	0	0
MDG	0	0	0	0	0	0	0	0
MNP	0	0	0	0	0	0	0	0
NER	0	0	0	0	0	0	0	0
NPL	0	0	0.1	0.1	0	0	0	0
PNG	0	0	0	0	0	0	0	0
PSE	0	0	0	0	0	0	0	0
PYF	0	0	0	0	0	0	0	0
RUS	0	0.2	0.3	0.5	0.1	0	0	0.1
SLB	0	0	0	0	0	0	0	0

SMR	0	0	0	0	0	0	0	0
SYR	0	0	0	0	0	0	0	0
TON	0	0	0	0	0	0	0	0
TUV	0	0	0	0	0	0	0	0
UGA	0	0	0	0	0	0	0	0
WSM	0	0	0	0	0	0	0	0
BRA	-0.1	0	-0.1	0	0	0	0	0
DZA	-0.1	-0.1	-0.1	0	0	0	0	0
MAC	-0.1	0.4	0.5	1	0.1	0	0	0.1
COL	-0.2	-0.1	-0.2	0	0	0	0	0.1
HTI	-0.3	-0.1	-0.3	-0.1	0	0	0	0
JOR	-0.3	-0.1	-0.3	-0.1	0	0	0	0
MNG	-0.3	-0.1	-0.3	-0.1	0	0	0	0
ZWE	-0.3	0	-0.1	0.2	0	0	0	0.1
BOL	-0.4	-0.1	-0.4	-0.1	0	0.1	0	0.1
CYM	-0.4	0	-0.1	0.3	0.1	0	0	0.1
KEN	-0.4	-0.1	-0.3	0	0	0.1	0	0.1
MNE	-0.4	0	-0.1	0.4	0.1	0	0	0.1
TGO	-0.4	-0.1	-0.3	-0.1	0	0	0	0.1
TTO	-0.4	-0.1	-0.3	-0.1	0	0	0	0.1
TZA	-0.4	-0.1	-0.2	0.1	0	0	0	0.1
ARM	-0.5	-0.1	-0.4	0	0	0.1	0	0.1
BFA	-0.5	-0.2	-0.5	-0.2	0	0.1	0	0.1
KNA	-0.6	-0.3	-0.6	-0.3	0	0.1	0	0.1
SUR	-0.6	-0.3	-0.6	-0.2	0	0.1	0	0.1
URY	-0.6	-0.1	-0.3	0.1	0.1	0.1	0	0.1
YEM	-0.6	0.8	0.9	2.3	0.4	0.1	0	0.4
GUY	-0.7	-0.3	-0.7	-0.3	0	0.1	0	0.1
KAZ	-0.7	-0.3	-0.6	-0.2	0	0.1	0	0.1
BWA	-0.8	-0.2	-0.5	0.1	0.1	0.1	0	0.2
CMR	-0.8	-0.3	-0.7	-0.2	0	0.1	0	0.1
MRT	-0.8	-0.3	-0.7	-0.2	0	0.1	0	0.1
IRN	-1	-0.4	-1	-0.4	0	0.1	0	0.1
QAT	-1	-0.3	-0.8	-0.1	0.1	0.1	0	0.2
BRN	-1.1	-0.3	-0.8	-0.1	0.1	0.1	0	0.2
SDN	-1.1	-0.5	-1.1	-0.5	0	0.2	0	0.2
ABW	-1.2	-0.5	-1.2	-0.5	0	0.2	0	0.2
ARE	-1.2	0.1	-0.3	1	0.3	0.1	0	0.3
ETH	-1.3	-0.5	-1.2	-0.5	0.1	0.2	0	0.2
MOZ	-1.3	-0.2	-0.7	0.4	0.2	0.1	0	0.3
CHL	-1.4	-0.5	-1.3	-0.4	0.1	0.2	0	0.2
KGZ	-1.4	-0.5	-1.3	-0.4	0.1	0.2	0	0.2
TJK	-1.4	-0.4	-1.1	-0.1	0.1	0.2	0	0.2
BHR	-1.5	-0.4	-1.2	-0.1	0.1	0.2	0	0.3
GTM	-1.6	-0.5	-1.2	-0.1	0.1	0.2	0	0.3

PAN	-1.7	-0.7	-1.6	-0.6	0.1	0.2	0.1	0.3
USA	-1.8	-0.5	-1.2	0.1	0.1	0.2	0	0.4
DOM	-2.2	-0.9	-2.1	-0.7	0.1	0.3	0.1	0.4
LBN	-2.2	-0.9	-2	-0.7	0.1	0.3	0.1	0.3
ISR	-2.6	-0.9	-2.2	-0.5	0.1	0.3	0.1	0.4
KWT	-2.6	-1.1	-2.5	-1.1	0.1	0.4	0.1	0.4
CUW	-3	-1.3	-3	-1.3	0.1	0.4	0.1	0.4
SAU	-3.1	-1.1	-2.7	-0.7	0.1	0.4	0	0.5
BLR	-3.2	-0.5	-1.9	0.7	0.4	0.4	0.1	0.7
THA	-3.3	0	-1	2.3	0.7	0.3	0	0.9
MUS	-3.6	-1.3	-3.2	-0.9	0.2	0.5	0.1	0.6
MDV	-3.7	-1.3	-3.2	-0.8	0.2	0.5	0.1	0.6
SLV	-3.7	0.1	-1.1	2.6	1.1	0.6	1	1.1
BRB	-4	-1.8	-4	-1.8	0.1	0.6	0.1	0.6
BLZ	-4.1	-1.8	-4.1	-1.8	0.1	0.6	0.1	0.6
SGP	-4.2	-1.2	-3.2	-0.3	0.3	0.5	0.1	0.7
GRL	-4.7	-2.1	-4.7	-2.1	0.2	0.7	0.2	0.7
MEX	-5	-2	-4.6	-1.6	0.2	0.6	0.1	0.7
EU	-5.7	-1.8	-4.3	-0.4	0.6	0.6	0.2	0.9
NIC	-6.3	-2.5	-5.9	-2.1	1	1	1	1.1
MKD	-6.6	-2.1	-5.4	-0.9	2.4	1.5	2.4	1.7
VCT	-6.6	-2.7	-6.4	-2.5	1.3	1.2	1.3	1.2
IRQ	-7.4	-3.2	-7.4	-3.2	1.9	1.5	1.9	1.5
PHL	-7.8	-2.6	-6.5	-1.3	4.2	2.3	4.2	2.4
TUN	-8.1	-3.4	-7.9	-3.2	2.9	1.9	2.9	1.9
FJI	-8.2	-3.3	-7.7	-2.8	3.1	1.8	3.1	1.9
KHM	-8.8	-3.6	-8.4	-3.3	4.1	2.4	4.1	2.4
NZL	-8.9	-3.2	-7.7	-2.1	5.9	3	5.9	3.1
JAM	-9	-3.9	-8.9	-3.9	3.8	2.3	3.8	2.3
CRI	-9.3	-3.4	-8.3	-2.4	6.7	3.4	6.6	3.5
CHE	-9.9	-4.4	-9.9	-4.3	5.3	3	5.3	3
ISL	-10.7	-4.7	-10.7	-4.7	6.9	3.6	6.9	3.6
MHL	-10.7	-4.7	-10.7	-4.7	6.9	3.6	6.9	3.6
TKM	-10.7	-4.7	-10.7	-4.7	6.9	3.6	6.9	3.6
GBR	-12.3	-5.6	-12.2	-5.4	0.5	1.4	0.4	1.5
AUS	-13.2	-5.2	-12.3	-4.2	0.3	1.8	0.1	2
JPN	-21.8	-9.5	-21.5	-9.2	0.2	3	0.1	3.1

Table S7 – Expected virgin content (VWG/TWG), improved recycling scenarios.

Scenario	Scenario code	Mean	Lower coverage bound	Upper coverage bound
baseline	B.1	0.916	0.896	0.936
increase sorting	R.1	0.887	0.871	0.902

increase sorting & yield	R.2	0.869	0.858	0.880
50%, mechanical	R.3	0.741	0.688	0.793
50%, chemical	R.4	0.730	0.670	0.789
increase mech, chem, yield	R.5	0.609	0.608	0.610

S2 – Definitions

Many of the terms used in this paper have ambiguous definitions, so we define them below as we used them for modeling purposes. This list also includes parameter definitions unique to our MCMFA model.

Mismanaged waste: waste that is inadequately managed (e.g., open, uncontrolled landfills) such that it has a high likelihood of leaking into the environment. This is the same definition from Jambeck's 2010 analysis of plastic inputs into the ocean. [5]

Recycling rate: The proportion of generated waste that is collected for both domestic and foreign recycling.

Sorting efficiency: The proportion of waste inputs to material recovery facilities that are sent for further processing at recycling facilities.

Reprocessing efficiency: The yield of mechanical or chemical recycling processes.

Recycling efficiency: The combined efficiency of sorting and reprocessing, calculated as the product of the two.

Chemical recycling fraction: The proportion of recycling that chemical recycling constitutes. This is zero for the baseline model, but we include it so we can compare the effectiveness of chemical and mechanical recycling.

Trade fraction: The proportion of waste that a country collects for recycling (product of total waste generation and recycling rate) that is exported for foreign recycling.

Import recycling rate: The proportion of imported waste that is sent to recycling facilities

Import mismanagement rate: The proportion of disposed imported waste that is mismanaged.

Total waste generation (TWG): The total quantity of waste produced in a year, including waste made of virgin and recycled plastic. TWG is directly observable and therefore measured, but it is not equal to the rate at which waste enters the system because it includes waste containing recyclate (which is waste that entered the system in the past and has been disposed of again). This is the sum of the throughputs of states G1, G2, and G3.

Virgin waste generation (VWG): The quantity of virgin waste produced in a year, i.e., the rate at which plastic waste enters the system through G1 and leaves through the sink states representing permanent disposal. We use VWG and linear disposal interchangeably, as they are equivalent. Note that recycling does offset VWG, unlike TWG.

Table S8 – Plastic recycling rate data sources

Country	Plastic recycling rate (%)	Source
Argentina	11	Veolia Group 2018.[6]
Australia	14	Veolia Group 2018.[6]
Bahrain	10	Veolia Group 2018.[6]
Brazil	19	Veolia Group 2018.[6]
Canada	9	Environment and Climate Change Canada 2019.[7]
China	25	Veolia Group 2018.[6]
European Union	30	Deloitte Sustainability 2017.[8]
Hong Kong	25	Veolia Group 2018.[6]
Indonesia	10	Luhut B. Pandjaitan, World Economic Forum Article. 2020.[9]
Japan	20	Jain, Amit 2020.[10]
Korea, Rep.	23	Statista 2019.[11]
Malaysia	24	The World Bank Group 2021.[12]
Mexico	24	Veolia Group 2018.[6]
Morocco	25	Veolia Group 2018.[6]
Saudi Arabia	10	Veolia Group 2018.[6]
Singapore	20	Jain, Amit 2020.[10]
South Africa	20	Veolia Group 2018.[6]
Thailand	18	The World Bank Group 2021.[13]
United Kingdom	32	British Plastics Federation 2022.[14]
United States	9	US Environmental Protection Agency.[15]

Table S9 – Table of data sources.

Data	Source	Source type
Mismanagement rates	Law et al.[1]	Literature study
Total waste generation	World Bank,[2] Law et al.[1]	Report, literature study
Recycling rates	*Many, see table S8*	*Many, see table S8*
Sorting and reprocessing efficiency	Antonopoulos et al.[3]	Literature study
Trade flows	CEPII-BACI [4]	Database
Import recycling and mismanagement rates	Law et al.[1]	Literature study

References

1. Law, K. L.; Starr, N.; Siegler, T. R.; Jambeck, J. R.; Mallos, N. J.; Leonard, G. H. The United States' contribution of plastic waste to land and ocean. *Science Advances* **2020**, *6* (44), eabd0288. DOI: doi:10.1126/sciadv.abd0288.
2. Kaza, S., Lisa Yao, Perinaz Bhada-Tata, and Frank; Woerden, V. *What a Waste 2.0: A Global Snapshot of Solid Waste Management to 2050*; Urban Development Series.; World Bank, 2018. DOI: 10.1596/978-1-4648-1329-0.
3. Antonopoulos, I.; Faraca, G.; Tonini, D. Recycling of post-consumer plastic packaging waste in the EU: Recovery rates, material flows, and barriers. *Waste Management* **2021**, *126*, 694-705. DOI: https://doi.org/10.1016/j.wasman.2021.04.002.
4. BACI: International Trade Database at the Product-Level. The 1994-2007 Version. *BACI*. October 2010. http://www.cepii.fr/CEPII/fr/publications/wp/abstract.asp?NoDoc=2726 (accessed May 2021).
5. Jambeck, J. R.; Geyer, R.; Wilcox, C.; Siegler, T. R.; Perryman, M.; Andrady, A.; Narayan, R.; Law, K. L. Plastic waste inputs from land into the ocean. *Science* **2015**, *347* (6223), 768-771. DOI: doi:10.1126/science.1260352.
6. Plastic recycling: a key link in the circular economy. 2018. available online at: https://www.planet.veolia.com/en/plastic-recycling (Accessed, June 5, 2022)
7. Canada, E. a. C. C. *Economic Study of the Canadian Plastic Industry, Markets, and Waste*; 2019. https://publications.gc.ca/site/archivee-archived.html?url=https://publications.gc.ca/collections/collection_2019/eccc/En4-366-1-2019-eng.pdf.
8. (8) Sustainability, D. *Blueprint for plastics packaging waste: Quality sorting & recycling*; 2017. https://www2.deloitte.com/content/dam/Deloitte/my/Documents/risk/my-risk-blueprint-plastics-packaging-waste-2017.pdf. (Accessed, June 5, 2022).
9. (9) Pandjaitan, L. B. *Here's how Indonesia plans to take on its plastic pollution challenge*. World Economic Forum, 2020. https://www.weforum.org/agenda/2020/01/here-s-how-indonesia-plans-to-tackle-its-plastic-pollution-challenge/ (Accessed, June 5, 2022).
10. Jain, A. State of Plastics Waste in Asia and the Pacific- Issues, Challenges and Circular Economic Opportunities. In *10th Regional 3R and Circular Economy Forum in Asia and the Pacific* UNITED NATIONS CENTRE FOR REGIONAL DEVELOPMENT: 2020, Discussion paper available online at :

https://sdgs.un.org/sites/default/files/2020-12/UNCRD_10th%203R%20Forum_Webinar%20III-Background%20paper-FINAL-on%20Report.pdf (accessed June 5, 2022).
11. Yoon, J. S. *Plastic waste disposal South Korea 2017, by method*. Statista, 2019. https://www.statista.com/statistics/1100696/south-korea-plastic-waste-disposal-by-method/ (accessed June 5, 2022).
12. World Bank Group. *Market Study for Malaysia: Plastics Circularity Opportunities and Barriers*; Washington DC, 2021, available online at: https://www.worldbank.org/en/country/malaysia/publication/market-study-for-malaysia-plastics-circularity-opportunities-and-barriers, accessed June 5, 2022)
13. World Bank Group. *Market Study for Thailand: Plastics Circularity Opportunities and Barriers*; Washington DC, 2021. https://www.worldbank.org/en/country/thailand/publication/market-study-for-thailand-plastics-circularity-opportunities-and-barriers, (accessed June 5, 2022).
14. Federation, B. P. *Plastic Recycling*. 2022. https://www.bpf.co.uk/sustainability/plastics_recycling.aspx#s5 accessed June 5, 2022).
15. US EPA. *Advancing Sustainable Materials Management: Facts and Figures Report*. 2020. https://www.epa.gov/facts-and-figures-about-materials-waste-and-recycling/advancing-sustainable-materials-management (accessed June 5, 2022).
